# High Prevalence of *Helicobacter pylori* Infection in Special Needs Schools in Japan

**DOI:** 10.3389/fped.2021.697200

**Published:** 2021-07-08

**Authors:** Toshihiko Kakiuchi, Ayako Takamori, Muneaki Matsuo

**Affiliations:** ^1^Department of Pediatrics, Faculty of Medicine, Saga University, Saga, Japan; ^2^Clinical Research Center, Saga University Hospital, Saga, Japan

**Keywords:** *Helicobacter pylori*, developmental disorders, adolescents, special needs schools, Japan

## Abstract

**Background:** Developmental disorders and high *Helicobacter pylori* (*H. pylori*) infection rates have been reported. This study aimed to examine the prevalence of *H. pylori* in a special needs school where all students had developmental disorders in Japan.

**Methods:** In 2017, third-grade junior high school and second- and third-grade high school students attending a special needs school with developmental disorders were enrolled. Participants of Saga Prefecture's *H. pylori* test and treat project, which comprised third-grade junior high school students not from special needs school, were assigned to the control group.

**Results:** In the control group, *H. pylori* positive results were 3.18% (228/7,164) students. Similarly, in developmental disorder group, *H. pylori* positive results were 6.80% (13/191) students. For the developmental disorder and control groups, this present examination sensitivity was 7.03% (13/185), specificity was 96.76% (6,815/7,043), positive predictive value was 5.39% (13/241), negative predictive value was 97.54% (6,815/6,987), Likelihood ratio of a positive result 2.17 and Odds ratio was 2.26 (95% confidence interval: 1.27–4.03, *p* = 0.005).

**Conclusion:** The prevalence of *H. pylori* infection was significantly higher in adolescents with developmental disorders than in typically developing adolescents.

## Introduction

*Helicobacter pylori* (*H. pylori*) infects the human gastric mucosa and causes gastritis and gastric cancer (1, 2). *H. pylori* infection also affects aspects of stomach function, such as gastric acid secretion, which causes changes in the stomach environment and intestinal microbiome (3, 4).

The global *H. pylori* incidence in children varies significantly, from 2.5% in Japan to 34.6% in Ethiopia (5), and it is greatly affected by economic power and living environment of the country (6). The primary modes of transmission are thought to be fecal-oral and oral-oral, but some indirect evidence has also been published for transmission via drinking water and other environmental sources (7). The patterns of spreading of *H*. *pylori* under conditions of high prevalence differ from those in developed countries (8).

Due to the suspicion of an association between *H. pylori* infection in early life and neurodevelopmental problems (9, 10), studies have been conducted overseas to determine the association between them, but there are no data for Japan (11). Therefore, the current study

aimed to examine the incidence of *H. pylori* infection in Japan in patients with developmental disorders in comparison with the control group and to discuss the association between *H. pylori* and developmental disorders.

## Methods and Materials

### Study Design and Subjects

Saga Prefecture is one of the local governments in Japan, and its population is about 830,000. There are 108 junior high schools (including eight special needs schools), with a total of ~8,500 students in the third grade. In the spring of 2016, we initiated a program for screening and treatment of *H. pylori* infection in all third-grade junior high school students, including special needs schools, in Saga Prefecture (approved by the institutional review board of Saga University Hospital [approval number: 2015-12-19]) (12). In 2017, third-grade junior high school students of special needs school with a developmental disorder, including intellectual disabilities, autism spectrum disorder, attention-deficit hyperactivity disorder, and learning disabilities, were eligible for participation. Similarly, in addition to the Saga Prefecture project, second- and third-grade high school students of special needs school with a developmental disorder were also enrolled (developmental disorder group). The control group comprised third-grade junior high school students not from a special needs school in the Saga Prefecture project.

### Testing for *H. pylori*

After obtaining written informed consent from each student and his or her guardian, urine samples were screened for the presence of anti–*H. pylori* immunoglobulin G antibody by immunochromatography (RAPIRAN® Otsuka Pharmaceutical Co., Ltd., Tokyo, Japan). The diagnostic sensitivity and specificity of the urinary test have been reported to be 89 and 93%, respectively (13, 14). Students who screened positive on the urine antibody test also received an *H. pylori* stool antigen detection kit (Testmate Rapid Pylori Antigen® Wakamoto Pharmaceutical Co., Ltd., Tokyo, Japan) to check for the presence or absence of *H. pylori* infection (15). In the present study, students positive for both *H. pylori* urinary antibody and fecal *H. pylori* antigen tests were defined as *H. pylori* infection. Students negative for *H. pylori* urinary antibody test were defined as *H. pylori* non-infection.

### Statistical Analysis

**Figure 1:**
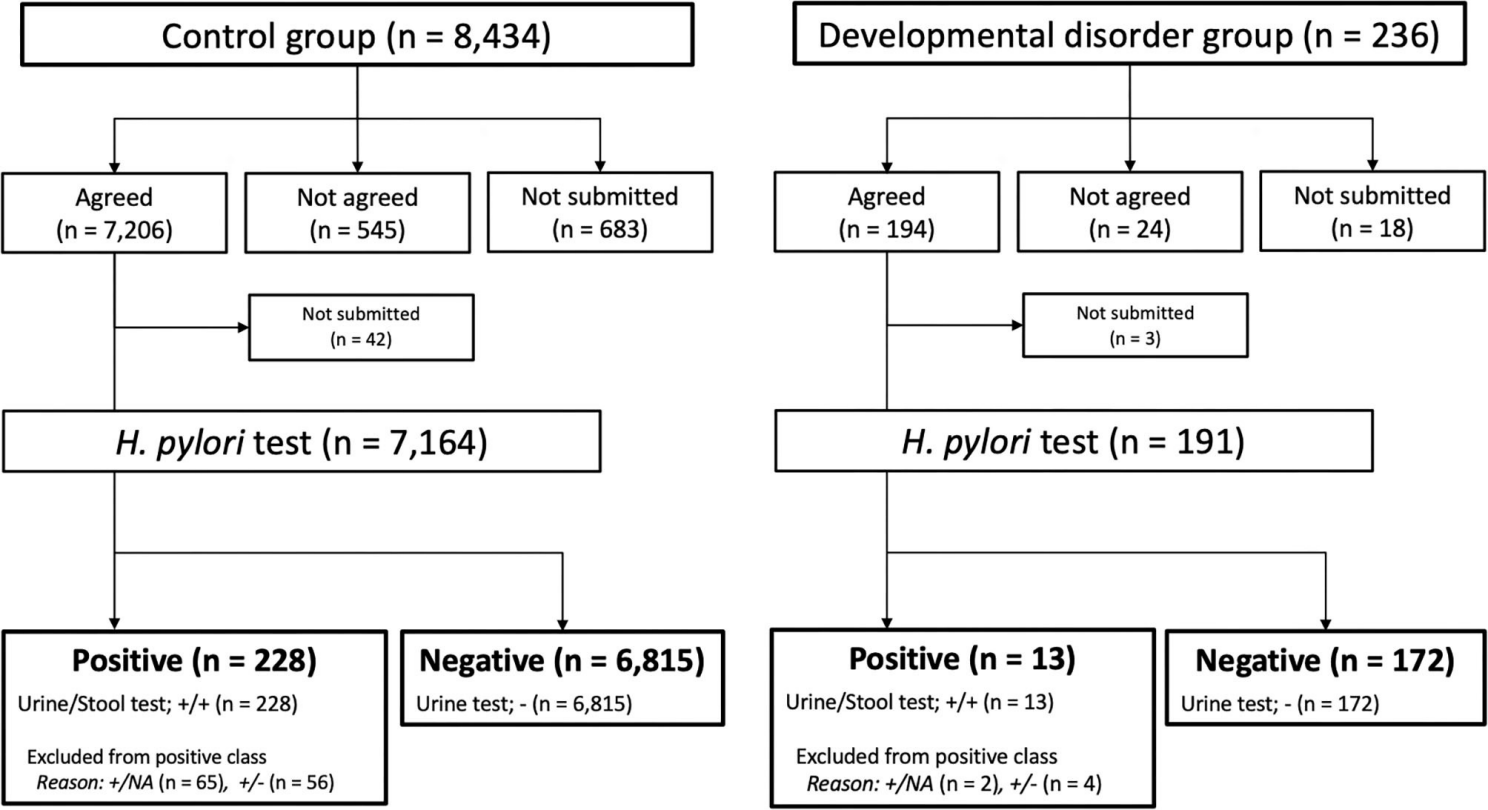
Participant's flowchart and *Helicobacter pylori* test results. *H. pylori, Helicobacter pylori*; NA, not available.

For the developmental disorder group and the control group, the comparative statistical analyses were conducted with contingency tables that summarized the results of the test classifiers examined. After the *H. Pylori* tests were examined, the patients were classified as follows: the positive was defined urine (+) and stool (+) test patients (=*H. pylori* infection patients), and the negative was defined urine (-) test patients (=*H. pylori* non-infection patients) (See Figure 1).

The parameters were estimated and shown as sensitivity, specificity, positive predictive value, negative predictive value, the likelihood ratio of a positive result, and the odds ratio. And chi-square tests were applied. Statistical software JMP version 15.2.0 (SAS Institute Inc., Cary, NC, USA) was used. All *p* < 0.05 indicated statistical significance.

## Results

### Student Characteristics

Figure 1 summarizes the flowchart for the participants in both the control group and the developmental disorders group. There was no sex bias between the two groups. Among the 8,434 students in the control group, 7,206 agreed to participate in the Saga Prefecture project, and 7,164 received urine *H. pylori* antibody tests. Among the 236 students in the developmental disorders group, 194 agreed to participate in both the Saga Prefecture project and our study, and 191 received urine *H. pylori* antibody tests.

### Test for *H. pylori* Infection Rate

Figure 1 shows the results of the urine *H. pylori* antibody and fecal *H. pylori* antigen tests in both groups. In the control group, positive results were 3.18% (228/7,164) students, 121 students with fecal *H. pylori* antigen tests negative or not implemented were excluded from the positive results. Negative results were 95.13% (6,815/7,164) students. Similarly, in developmental disorder group, positive results were 6.80% (13/191) students, 6 students with fecal *H. pylori* antigen tests negative or not implemented were excluded from the positive results. Negative results accounted for 90.05% (172/191) of the students.

Table 1 shows test results for the *H. pylori* infection rate between both groups. For the developmental disorder and control groups, this present examination sensitivity was 7.03% (13/185), specificity was 96.76% (6,815/7,043), positive predictive value was 5.39% (13/241), negative predictive value was 97.54% (6,815/6,987), the likelihood ratio of a positive result 2.17 and Odds ratio was 2.26 (95% confidence interval: 1.27–4.03, *p* = 0.005). The developmental disorder group was approximately twice as likely to show positive results for *H. pylori* infection as the control group.

**Table 1:** Test for *Helicobacter pylori* infection rate between the control group and developmental disorders group.

***H. pylori* test**	**Developmental disorder group**	**Control group**	**Total**
Positive	13	228	241
	(7.03%)	(3.24%)	
Negative	172	6,815	6,987
	(92.97%)	(96.76%)	
total	185	7,043	7,228
**Parameter**			
Sensitivity	P(Positive|D)		7.03%
Specificity	P(Negative|Control)		96.76%
PPV	P(D|Positive)		5.39%
NPV	P(Control|Negative)		97.54%
LR+	Sensitivity/(1-Specificity)		2.17
Odds ratio			2.26
			95%CI (1.27–4.03)
Chi-squared test			*P* = 0.005

*Positive included urine (+) and stool (+) test patients. Negative included urine (–) test patients (See Figure 1)*.

*PPV, Positive predictive value*.

*NPV, Negative predictive value*.

*LR+, Likelihood ratio of a positive result*.

## Discussion

This study showed that the prevalence of *H. pylori* infection was significantly higher in adolescents with developmental disorders than in typically developing adolescents in Japan.

Kitchens et al. reviewed the association between *H. pylori* and intellectual and developmental disabilities overseas (11). However, the papers they cited did not include Japanese data, and there was a possibility that various biases existed due to the large age range of the subjects. Our study is the first report that includes Japanese data. Because the age range was limited to adolescence, the number of subjects was large, and the subjects in both groups were living in the same area; thus, confounding factors seemed to be small in our study.

The reasons for the high incidence of *H. pylori* infection in adolescents with developmental disorders are unknown. Kitchens et al. reported that maladaptive behaviors exhibited by individuals who have intellectual disabilities and developmental disabilities can be considered as risk factors for *H. pylori* infection (11). Maladaptive behaviors exhibited by individuals who have developmental disorders can be considered as risk factors for *H. pylori* infection because *H. pylori* has been cultivated from vomitus, saliva, and feces (16).

Morad et al. concluded that residential living with shared living quarters and eating in the same utensils contributed to the risk factors for *H. pylori* infection in people with intellectual disabilities and developmental disabilities (17). Lambert et al. determined that the most important factor for *H. pylori* infection was the duration of institutionalization (18). In Japan, schools for children with disabilities often have dormitories that house students with developmental disorders. Group life may have an effect on increasing the infection rate of *H. pylori*, regardless of the presence of developmental disorders. Unfortunately, this study could not investigate whether they had lived in groups.

Karachallou et al. reported that *H. pylori* infection in early life may be an important risk factor for poor neurodevelopment (18). Several epidemiological studies reported that H. pylori-infected family members are the risk factor for pediatric infection with *H. pylori* (19, 20). Osaki et al. reported that mother-to-child transmission of *H. pylori* was demonstrated in 80% of patients while assessing the genomic profiles of *H. pylori* isolates from family members by multi-locus sequence typing (21).

Our study demonstrates higher rates of *H. pylori* infection among adolescents with developmental disorders. However, we should be cautious in discussing causative associations between developmental disorders and *H. pylori* infection. In Japan, the incidence of *H. pylori* infection declines with each generation (22), and this is true even among the young generation (12). On the other hand, developmental disorders have been increasing annually in Japan (23). These two facts are contradictory and may be grounds for denying the association between *H. pylori* infection and developmental disorders.

There are several limitations to the present study. First, the control group could have included students with developmental disorders, as we did not have any ways to check their medical or medication history in the control group. Second, background factors, such as life history, that could be risk factors for *H. pylori* infection could not be compared between the two groups. Similarly, we were not able to compare the number of family members the number of people living together in the institutions, and economic status between the two groups. Third, the criterion chosen for screening of *H. pylori* infection was a urinary antibody test (which is not a gold standard test). There is an established screening program for kidney diseases in Japan (including Saga Prefecture) targeting third-grade students in junior high schools. Given the full inclusivity of students during this test through simple urine examination, we used the established system to obtain urine samples to screen for *H. pylori* infection. Only students positive for *H. pylori* urinary antibody test underwent a secondary test, fecal *H. pylori* antigen test. There was a possibility that false negatives were included as some students defined as negative for *H. pylori* infection.

In conclusion, *H. pylori* infection rates were found to be significantly higher in adolescents with developmental disorders than in typically developing adolescents in Japanese. Future Basic and clinical studies are needed to elucidate the direct relationship between *H. pylori* infection and developmental disorders.

## Data Availability Statement

The original contributions presented in the study are included in the article/supplementary material, further inquiries can be directed to the corresponding author/s.

## Ethics Statement

The ethical aspects of this study were reviewed and approved by the institutional review board of Saga University Hospital (approval number: 2016-12-03). Written informed consent to participate in this study was provided by the participants' legal guardian/next of kin.

## Author Contributions

TK and MM conceptualized, analyzed data, and prepared the manuscript. TK collected the data. AT performed statistical analyses. MM critically reviewed the manuscript. All authors approved the final version of the article, including the authorship list.

## Conflict of Interest

The authors declare that the research was conducted in the absence of any commercial or financial relationships that could be construed as a potential conflict of interest.

## Abbreviations

*H. pylori*: *Helicobacter pylori*.

## References

[1] Suzuki H Mori H. World trends for *H. pylori* eradication therapy and gastric cancer prevention strategy by *H. pylori* test-and-treat. J Gastroenterol. (2018) 53:354–61. 10.1007/s00535-017-1407-1 29138921 PMC5847180

[2] Uemura N Okamoto S Yamamoto S Matsumura N Yamaguchi S Yamakido M. *Helicobacter pylori* infection and the development of gastric cancer. N Engl J Med. (2001) 345:784–9. 10.1056/NEJMoa001999 11556297

[3] Llorca L Perez-Perez G Urruzuno P Martinez MJ Iizumi T Gao Z. Characterization of the gastric microbiota in a pediatric population according to *Helicobacter pylori* status. Pediatr Infect Dis J. (2017) 36:173–8. 10.1097/INF.0000000000001383 27820723

[4] Oh B Kim BS Kim JW Kim JS Koh SJ Kim BG. The effect of probiotics on gut microbiota during the *helicobacter pylori* eradication: randomized controlled trial. Helicobacter. (2016) 21:165–74. 10.1111/hel.12270 26395781

[5] Mišak Z Hojsak I Homan M. Review: *Helicobacter pylori* in pediatrics. Helicobacter. (2019) 24:e12639. 10.1111/hel.12639 31486243

[6] Zebala Torrres B Luceno Y Lagomarcino AJ Orellance-Manzano A George S Torres JP. Review: prevalence and dynamics of *Helicobacter pylori* infection during childhood. Helicobacter. (2017) 22:e12399. 10.1111/hel.12399 28643393

[7] Schwarz S Morelli G Kusecek B Manica A Balloux F Owen RJ. Horizontal versus familial transmission of *Helicobacter pylori*. PLoS Pathog. (2008) 4:e1000180. 10.1371/journal.ppat.1000180 18949030 PMC2563686

[8] Yucel O. Prevention of *Helicobacter pylori* infection in childhood. World J Gastroenterol. (2014) 20:10348–54. 10.3748/wjg.v20.i30.10348 25132751 PMC4130842

[9] Karachaliou M Chatzi L Michel A Kyriklaki A Kampouri M Koutra K. *Helicobacter pylori* seropositivity and childhood neurodevelopment, the rhea birth cohort in Crete, Greece. Paediatr Perinat Epidemiol. (2017) 31:374–84. 10.1111/ppe.12374 28640520

[10] Lee A. Early influences and childhood development. Does helicobacter play a role? Helicobacter. (2007) 12(Suppl. 2):69–74. 10.1111/j.1523-5378.2007.00567.x 17991180

[11] Kitchens DH Binkley CJ Wallace DL Darling D. *Helicobacter pylori* infection in people who are intellectually and developmentally disabled: a review. Spec Care Dentist. (2007) 27:127–33. 10.1111/j.1754-4505.2007.tb00334.x 17972442

[12] Kakiuchi T Matsuo M Endo H Nakayama A Sato K Takamori A. A *Helicobacter pylori* screening and treatment program to eliminate gastric cancer among junior high school students in Saga Prefecture: a preliminary report. J Gastroenterol. (2019) 54:699–707. 10.1007/s00535-019-01559-9 30770975

[13] Okuda M Kamiya S Journala M Kikuchi S Osaki T Hiwatani T. Diagnostic accuracy of urine-based kits for detection of *Helicobacter pylori* antibody in children. Pediatr Int. (2013) 55:337–41. 10.1111/ped.12057 23360308

[14] Yamamoto T Ishii T Kawakami T Sase Y Horikawa C Aoki N. Reliability of urinary tests for antibody to *Helicobacter pylori* in patients with pulmonary tuberculosis. World J Gastroenterol. (2005) 11:412–4. 10.3748/wjg.v11.i3.412 15637756 PMC4205350

[15] Shimoyama T. Stool antigen tests for the management of *Helicobacter pylori* infection. World J Gastroenterol. (2013) 19:8188–91. 10.3748/wjg.v19.i45.8188 24363508 PMC3857440

[16] Parsonnet J Shmuely H Haggerty T. Fecal and oral shedding of *Helicobacter pylori* from healthy infected adults. JAMA. (1999) 282:2240–5. 10.1001/jama.282.23.2240 10605976

[17] Morad M Merrick J Nasri Y. Prevalence of *Helicobacter pylori* in people with intellectual disability in a residential care centre in Israel. J Intellect Disabil Res. (2002) 46:141–3. 10.1046/j.1365-2788.2002.00382.x 11869384

[18] Lambert JR Lin SK Sievert W Nicholson L Schembri M Guest C. High prevalence of *Helicobacter pylori* antibodies in an institutionalized population: evidence for person-to-person transmission. Am J Gastroenterol. (1995) 90:2167–71. 8540509

[19] Goodman KJ Correa P. Transmission of *Helicobacter pylori* among siblings. Lancet. (2000) 355:358–62. 10.1016/S0140-6736(99)05273-3 10665555

[20] Vincent P Gottrand F Pernes P Husson MO Lecomte-Houcke M Turck D. High prevalence of *Helicobacter pylori* infection in cohabiting children. Epidemiology of a cluster, with special emphasis on molecular typing. Gut. (1994) 35:313–6. 10.1136/gut.35.3.313 8150338 PMC1374581

[21] Osaki T Konno M Yonezawa H Hojo F Zaman C Takahashi M. Analysis of intra-familial transmission of *Helicobacter pylori* in Japanese families. J Med Microbiol. (2015) 64:67–73. 10.1099/jmm.0.080507-0 25351712

[22] Miyamoto R Okuda M Lin Y Murotani K Okumura A Kikuchi S. Rapidly decreasing prevalence of *Helicobacter pylori* among Japanese children and adolescents. J Infect Chemother. (2019) 25:526–30. 10.1016/j.jiac.2019.02.016 31003956

[23] Williams JG Higgins JP Brayne CE. Systematic review of prevalence studies of autism spectrum disorders. Arch Dis Child. (2006) 91:8–15. 10.1136/adc.2004.062083 15863467 PMC2083083

